# Exploring Osmotic Dehydration for Food Preservation: Methods, Modelling, and Modern Applications

**DOI:** 10.3390/foods13172783

**Published:** 2024-08-31

**Authors:** Alexandra Mari, Danai Nikoleta Parisouli, Magdalini Krokida

**Affiliations:** School of Chemical Engineering, National Technical University of Athens, 15780 Athens, Greece

**Keywords:** osmotic dehydration, mass transfer, mathematical modelling, osmotic agents, kinetics, process parameters

## Abstract

This study summarizes the most recent findings on osmotic dehydration, a crucial step in food preservation. The many benefits of osmotic dehydration are listed, including longer shelf life and preserved nutritional value. Mass transfer dynamics, which are critical to understanding osmotic dehydration, are explored alongside mathematical models essential for comprehending this process. The effect of osmotic agents and process parameters on efficacy, such as temperature, agitation and osmotic agent concentration, is closely examined. Pre-treatment techniques are emphasized in order to improve process effectiveness and product quality. The increasing demand for sustainability is a critical factor driving research into eco-friendly osmotic agents, waste valorization, and energy-efficient methods. The review also provides practical insights into process optimization and discusses the energy consumption and viability of osmotic dehydration compared to other drying methods. Future applications and improvements are highlighted, making it an invaluable tool for the food industry.

## 1. Introduction

Reducing food waste is of particular importance for the environment and the economy. Therefore, it is necessary to employ methods to increase the shelf life of food and ensure its quality. The most common method in the food industry for this purpose is the reduction of food moisture through dehydration and drying [1]. Reducing the moisture content of food, below a specific limit, results in the inhibition of microorganisms and enzymatic activity, thus reducing water activity and extending the shelf life [2]. Also, the reduction of weight and volume, the reduction of transport, distribution, and storage costs, the ease of use by the consumer, the preservation of taste, smell, and nutritional value, rapid hydration, and good organoleptic characteristics are factors that reinforce the importance of dehydrating food [3].

Drying, the removal of water from an object regardless of the method, encompasses a variety of drying methods with different advantages, depending on the context of their use [4]. Condensation, the removal of water from a liquid, resulting in a thicker liquid, is primarily used in the preservation of milk, juices, and the production of confectionery concentrates. This process is carried out through evaporation, membranes, or freezing [5]. As far as solid foods are concerned, dehydration is the predominant technique, involving the removal of water through mechanical means and the application of artificial heat under controlled conditions of temperature, humidity and air flow. This method is particularly applied to the processing of fruits, vegetables, and meats and includes many various approaches [6].

Dehydration of foods can be achieved by several methods, including sun dehydration, hot air, microwaves, vacuum, and osmotic dehydration. The basic and simplest method widely applied until now is dehydration with hot air (convective drying) [7]. However, the quality characteristics of fruits and vegetables are negatively affected by this method—chemically, physically, and organoleptically—due to the increase in temperature [8]. Consequently, in recent years, there has been a strong interest in non-thermal methods, such as osmotic dehydration and dehydration using ultrasound and microwaves, for the production of innovative food products [9]. These methods not only contribute to the creation of products with better quality and organoleptic characteristics but also reduce the time and cost of processing [10].

Osmotic dehydration is an alternative, non-thermal dehydration method based on the principle of osmotic dehydration. By leveraging the natural tendency of water molecules to migrate from areas of lower solute concentration to higher, moisture is extracted from the food matrix. It involves soaking foods in a salt or sugar solution under ambient or modified ambient conditions in order to reduce their moisture content before further processing [11]. It is usually followed by other processes such as air dehydration, freeze-drying, and other drying processes in order to achieve better end product quality [12]. Osmotic dehydration leads the way, as it can reduce the weight of fresh fruits and vegetables by up to 50% while helping to reduce processing time and energy [13]. Osmotic dehydration is highly relevant due to the necessity of minimizing energy inputs for food preparation and the pursuit of sustainable solutions. The success is due to the lack of need for water phase transformation, which can be largely removed at moderate temperatures [14].

The objective of this study is to comprehensively examine the current state of knowledge in the field of osmotic dehydration research, providing a detailed summary of significant discoveries, approaches, and recent developments. The review aims to identify gaps in the existing understanding and highlight areas where further research is needed to advance the field. By summarizing recent findings, this study offers practical insights into the osmotic dehydration process, including considerations of energy consumption and potential future applications in the food industry. This holistic examination seeks to provide a foundation for future research, enabling advancements that can optimize the osmotic dehydration process and enhance its applicability and efficiency in industrial settings.

## 2. Mechanism of Osmotic Dehydration

**Figure 1:**
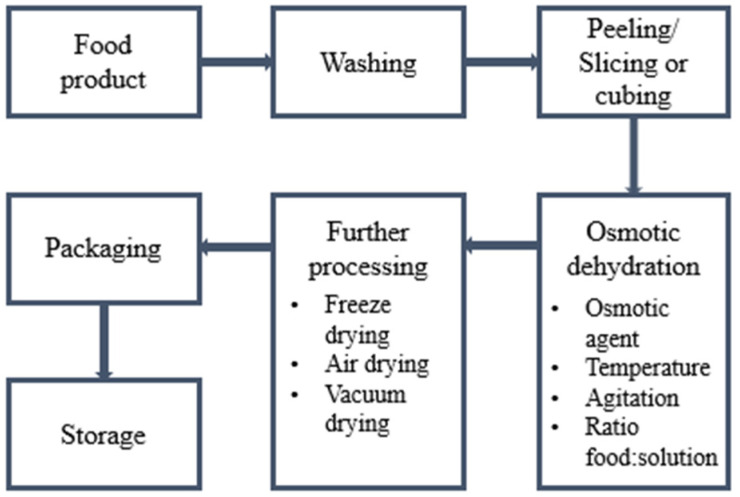
Flow chart of osmotic dehydration.

Osmotic dehydration, also called a “dewatering impregnation soaking process”, involves immersing solid food with a high-water content in an aqueous solution containing a high concentration of salts or sugars. This osmotic solution is characterized by a higher osmotic pressure and lower water activity compared to the food. The difference in osmotic pressure is the primary driver of mass transfer phenomena between food and solution [15]. The surface of the food, and more specifically, the cell wall, acts as a semi-permeable membrane. Thus, equilibrium conditions are maintained on both sides of the membrane by transferring water from the solution with a lower solute concentration (i.e., from the food) to the solution with a higher concentration [16]. The process of osmotic dehydration is presented in Figure 1.

Osmotic dehydration is a counter-current mass transfer process wherein the solute enters the food while moisture is eluted from the inside of the food into the hypertonic solution [17]. The first-layer cells in a cellular solid material submerged in a hypertonic solution contract as a result of water transfer due to the concentration gradient between the cells and the hypertonic solution. This creates a “chemical potential difference of water” between the first and second layers of cells as the first layer’s cells start to lose water. The cells in the second layer then start to plump up water to the cells in the first layer before contracting. As a function of operating time, the mass transfer and tissue shrinkage phenomena propagate from the material’s surface to its centre. After prolonged solid–liquid contact, the cells at the material centre eventually lose water, and the mass transfer mechanism tends toward equilibrium [18]. During the osmotic dehydration process, mass transfer and tissue shrinkage occur simultaneously [13]. However, the semi-permeable cell membrane is not selective, allowing soluble substances, such as organic acids, minerals, aromas, and pigments, to migrate from the food into the solution alongside water [19]. This transport is quantitatively negligible but significant in terms of product composition. Generally, the process is slow and largely depends on the permeability of cell membranes and cell architecture [20]. The osmotic pressure difference between the food and the hypertonic solution provides the necessary driving force for water movement from the food to the osmotic solution. The complex cellular structure of the biological material acts as a barrier to water diffusion [17]. Moisture removal primarily occurs through capillary flow and diffusion, while leaching and solute uptake occur only by diffusion [20]. These mass exchanges between the food and the hypertonic solution can impact the overall quality and quantity of the dehydrated products [17]. The semipermeable nature of plant tissues and the smaller molecular size of water molecules facilitate movement of water from the food. This results in a decrease in moisture content of up to 50% of the weight of fresh fruits and vegetables over time until the equilibrium state is reached [12]. The result of solid gain and water loss is the creation of intermediate moisture products with reduced water activity, significantly inhibiting chemical, biological and physical processes that degrade food, thereby extending shelf life [12].

**Figure 2:**
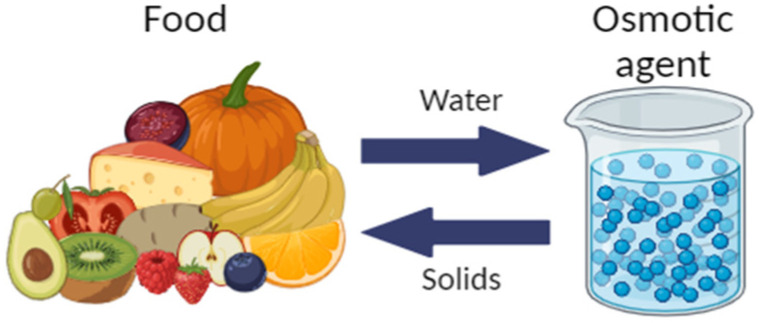
Mechanism of osmotic dehydration (Created by BioRender.com).

The mass transfer phenomenon is presented in Figure 2.

### 2.1. Mathematical Modelling of Osmotic Dehydration

The modelling of osmotic dehydration is based on the calculation of Water Loss (WL) and Solid Gain (SG). The numerical values of WL and SG are expressed on a dry basis and are calculated by Equations (1) and (2).

(1) $$WL=\frac{\left(M_{o}-m_{o}\right)-(M-m)}{m_{o}}$$

(2) $$SG=\frac{\left(m-m_{o}\right)}{m_{o}}$$

where *M*_0_ is the initial mass of the fresh food, *M* is the mass of the food after time *t* of osmotic treatment, *m* is the dry mass of the food after time *t* of osmotic treatment, and m_0_ is the dry mass of the fresh product [21].

There are two fundamental approaches to the mathematical description of osmotic dehydration: the macroscopic and the microscopic view. According to the macroscopic view, mechanisms observed at the cellular level are not taken into account; that is, it is assumed that the tissue of the food is homogeneous. In contrast, the microscopic view recognizes the heterogeneous properties of tissue and is based on the microstructure of the cell. This approach is complex but accurate [22].

Hence, the mass transfer phenomenon during osmotic dehydration is approached macroscopically, with the most widespread method of analysis based on Fick’s 2nd law of diffusion. This law describes the behaviour diffusion in a non-steady state. The phenomenon refers to the transfer of soluble materials from the surrounding space and vice versa. Therefore, if foods are considered porous solids, Fick’s law can provide satisfactory results. This relationship in the case of plate geometry is described by Equation (3).

(3) $$\frac{\partial C}{\partial t}=D_{e}\;\frac{\partial^{2}C}{\partial x^{2}}$$

where *C* is the concentration, *D_e_* is the diffusion coefficient, *x* is the characteristic distance of diffusion, and t is the time.

During the application of Fick’s 2nd law, several assumptions are made to simplify the complex process of osmotic dehydration. It is assumed that the concentration of the osmotic solution remains constant through the dehydration process, the surface resistance in comparison to the internal resistance is considered negligible, no shrinkage of the food occurs during osmotic dehydration, and the temperature is uniformly distributed [19].

A variety of mathematical models have been developed to describe osmotic dehydration [22]. Empirical, semi-empirical, and mechanistic are widely utilized due to their ability to be applied to non-classical geometries [23]. The kinetic variables of water loss, solids gain, and processing time are directly correlated in order to derive coefficients or factors that facilitate the interpretation of the physical process. Among these, the most commonly used empirical and semi-empirical models are further analysed below. These models play a crucial role in enhancing the understanding of osmotic dehydration and in the optimization of the process for various food products.

#### 2.1.1. Azuara’s Model

Using a mass balance to analyse the movement of water in the food during osmotic dehydration, ref. [24] developed a model to quantify the rate of water loss and solids gain as a function of time. This model resulted in equations characterized by two adjustable parameters, s_1_ or s_2_ and *WL*∞ or *SG*∞. The *WL* and *SG* are calculated by Equations (4) and (5).

(4) $$WL=\frac{s_{1}\cdot t\cdot WL\infty }{1+s_{1}\cdot t}$$

(5) $$SG=\frac{s_{2}\cdot t\cdot SG\infty }{1+s_{2}\cdot t}$$

Equations (4) and (5) can be linearized, into Equations (6) and (7) respectively.

(6) $$\frac{t}{WL}=\frac{1}{s_{1}\cdot WL\infty }+\frac{t}{WL\infty }$$

(7) $$\frac{t}{SG}=\frac{1}{s_{2}\cdot SG\infty }+\frac{t}{SG\infty }$$

The equilibrium water loss (*WL*∞) and solid gain (*SG*∞) are estimated from the slope and intercept of the plot of (*t*/*WL*) and (*t*/*SG*) versus t, using *WL* and *SG* values determined from the experimental data at different times.

#### 2.1.2. Peleg’s Model

Peleg (1988) developed a simple mathematical model that fitted sorption curves that approach the equilibrium asymptotically [25]. The equation expressing the model (Equation (8)) is described using two parameters, *k*_1_ and *k*_2_.

(8) $$M\left(t\right)=M_{o}+\frac{t}{k_{1}+k_{2}\cdot t}$$

The Peleg constant *k*_1_ relates to the initial rate of mass transfer, *t* = *t*_0_. The equilibrium moisture *M*_g_, according to this model, i.e., when *t*→∞, is given by Equation (9).

(9) $$M_{g}=M_{o}+\frac{1}{k_{2}}$$

Some researchers consider the water loss (WL) instead of the moisture, so Equation (10) is used and Equation (11) for the description of solid gain (SG).

(10) $$WL=\frac{t}{{k_{1}}^{W}+{k_{2}}^{W}\cdot t}$$

(11) $$SG=\frac{t}{{k_{1}}^{S}+{k_{2}}^{S}\cdot t}\;$$

#### 2.1.3. Page’s Model

Page (1949) discovered that the drying of a thin layer of food followed a straightforward exponential equation in a study to ascertain the impacts of the product’s initial moisture content, the drying air’s temperature, and its relative humidity [26]. The water loss and solid gain can be described by Equations (12) and (13).

(12) $$\frac{WL}{WL\infty }=1-\exp\left(-A_{w}\cdot t^{B_{w}}\right)\;$$

(13) $$\frac{SG}{SG\infty }=1-\exp\left(-A_{S}\cdot t^{B_{S}}\right)\;$$

where *A_W_* or *A_S_* is the dehydration constant and *B_W_* or *B_S_* is Page’s parameter. When this model is applied to the drying of a thin layer of material, only the *A_w_* parameter depends on the temperature, while the *B_W_* parameter remained constant.

#### 2.1.4. Panagiotou Model

The model that was developed by Panagiotou et al. (1998) describes mass transfer phenomena taking place during osmotic dehydration, and consists of first-order kinetics [27]. Water loss and solid gain are described by Equations (14) and (15).

(14) $$WL={WL}_{e\;}(1-\exp\left(-K_{WL}\cdot t\right)$$

(15) $$SG={SG}_{e\;}(1-\exp\left(-K_{SG}\cdot t\right)$$

The water loss and solid gain constants (K_WL_, K_SG_) are calculated by Equations (16) and (17).

(16) $$K_{WL}=a_{o}{\left(\frac{C}{C_{o}}\right)}^{a_{c}}{\left(\frac{T}{T_{o}}\right)}^{a_{T}}{\left(\frac{R}{R_{o}}\right)}^{a_{R}}$$

(17) $$K_{SG}=A_{o}{\left(\frac{C}{C_{o}}\right)}^{A_{c}}{\left(\frac{T}{T_{o}}\right)}^{A_{T}}{\left(\frac{R}{R_{o}}\right)}^{A_{R}}$$

The equilibrium values denoted as *WL_e_* and *SG_e_* in this model represent the values for water loss (*WL*) and solids gain (*SG*), respectively, after a prolonged processing time, specifically after 24 h of osmotic dehydration, when the time (*t*) approaches infinity (*t*→∞). These equilibrium values are critical for understanding the maximum extent of dehydration and solute incorporation achievable under specific conditions. The determination of the variables within the equations is achieved through linearization regression, based on least squares of the residuals—the differences between observed and calculated values—thus ensuring an optimal fit of the model to the experimental data.

#### 2.1.5. Crank’s Model

Crank put forth an equation for one-dimensional diffusion with an infinite number of solutions based on Fick’s second law (Equation (18)).

(18) $$(\frac{W_{LT}}{W_{L\infty }})=2{\left(\frac{Dt}{\pi l^{2}}\right)}^{\frac{1}{2}}$$

where *WLt*/*Xt* is the amount of water leaving (*WL*) or solute entering (*SG*) the food sample at time *t*, *WL*∞/*X*∞ is the amount of water leaving or solute solids entering the sample at infinite time (*WL*∞ or *SG∞*), *D* stands for the effective diffusion coefficient, and *L* represents half thickness of the slab [13].

### 2.2. Comparison of Models

The abovementioned models offer distinct methodologies for describing the phenomenon of osmotic dehydration. The food product’s unique properties, including its composition, structure, and water holding capacity, as well as the intended results of the osmotic dehydration process, determine which model is better. A short comparison is presented in Table 1.

Azuara’s model incorporates changeable parameters, providing flexibility in fitting experimental data to capture the rate of water loss and solids gain during the entire process without conducting the experiments for long duration. This model can characterize the OD of different food products, without geometrical restrictions or specific process conditions [24,28,29,30]. A limitation of Azuara’s model is its reliance on experimental data within a specific range to accurately evaluate mass transfer at equilibrium via linear regression, restricting its applicability outside of this range [31]. The Azuara model has demonstrated its efficacy in explaining the kinetics of water loss (WL) and solid gain (SG) during the osmotic dehydration of apples, banana, kiwifruit, cherry tomato, and goat meat [31], with correlation coefficients (R^2^) exceeding 0.90, underscoring its reliability.

Peleg’s model provides a fundamentally simple yet effective approach to depicting sorption curves as they approach equilibrium, sharing mathematical similarities with Azuara’s model. Using experimental data gathered over brief periods of time, both of these models can predict the kinetics of water sorption in food products, including determination of equilibrium moisture levels [31,32,33,34]. Peleg’s model has been successful in describing mass transfer kinetics during OD of apples [35], kiwifruit [36], potato [37] and goat meat [33], achieving R^2^ values higher than 0.90.

Page’s model provides a simple exponential formula that works well for a thin material layer. In particular, the model has been fitted in osmotic dehydration of apple, banana, cherry tomato, and kiwifruit [20].

Panagiotou’s first-order kinetics model takes into account a number of influencing variables, including temperature, concentration, and beginning conditions. As a result, it could be more suitable when considering multiple influencing factors. Panagiotou’s model demonstrates versatility in its applicability across various instances of osmotic dehydration and diverse food products. Specifically, the model has been used for the modelling of osmotic dehydration of sea bass fillets, beef meat [31], and carrots [38], with great efficiency.

Crank’s model, rooted in Fick’s second law, provides a theoretical foundation for understanding diffusion. However, it requires precise parameterization regarding thickness and diffusion coefficients. Its major limitation arises when experimental conditions significantly diverge from the model’s assumptions. Moreover, alterations in the physical properties of food induced by the process may lead to heightened diffusivities by disturbing the cell membrane, consequently enhancing cell permeability and facilitating mass transfer between the food and osmotic solution [39]. This model has been used to approximate the diffusion coefficient of water and solute within apple, banana, beetroot, and potato [20].

When designing an osmotic dehydration process, it is crucial to consider factors such as the nature of the food product, which includes its unique composition, structure, and water-holding capacity; the process conditions, including temperature, concentration of the osmotic solution, and initial conditions that significantly influence the dehydration process; and the desired product characteristics, like texture, flavour, and nutritional content, which depend on the mass transfer mechanisms. Empirical and semi-empirical models can help predict these outcomes, while the selection of the right model depends on the specific requirements and constraints of the process. For instance, Azuara’s model is flexible and widely applicable, whereas Crank’s model offers theoretical insights but requires precise conditions. Optimizing osmotic dehydration is a multifaceted task that requires a tailored approach to model selection. By considering the unique attributes of the food product, process parameters, and desired outcomes, one can accurately predict and control mass transfer phenomena, leading to enhanced food preservation, quality, and sensory properties. The selection of the optimal model requires an in-depth understanding of the underlying mass transfer mechanisms and the specific requirements of the osmotic dehydration procedure for a given food product. This strategic selection ensures the accurate prediction and optimization of the OD process, facilitating the achievement of desired product qualities and process efficiencies.

In the authors’ opinion, the selection of the most appropriate model for osmotic dehydration should be guided by the specific goals and constraints of the process. While empirical models such as Azuara’s and Peleg’s provide practical advantages due to their adaptability and simplicity, particularly when quick predictions are required, theoretical models like Crank’s offer a more in-depth understanding of the diffusion mechanisms at play, albeit with a greater demand for precise experimental conditions. The authors believe that an effective strategy involves balancing empirical ease with theoretical depth, taking into account the unique properties of the food product, the specific process parameters, and the desired final product characteristics. In some cases, combining elements from different models or customizing a single model may offer the best approach to accurately predict and optimize the osmotic dehydration process, ensuring that the intended quality and efficiency of the product are achieved.

## 3. Factors Affecting Osmotic Dehydration Process

Numerous variables, including the osmotic agents, duration, temperature, solute concentration, solution to sample ratio, agitation and material shape can affect osmotic dehydration.

### 3.1. Osmotic Agents

The osmotic dehydration process is significantly influenced by the physicochemical properties, molecular weight, solubility, and ionic state of the osmotic medium [40]. The osmotic medium serves as a driving force for the solute and water; therefore, it is of great importance in the osmotic dehydration process. Initially, an important factor is the desired organoleptic and physical properties of the final product [14]. Additionally, especially for industrial production, the cost of the osmotic agent is a necessary consideration [41]. An equally important factor is the molecular weight of the solvent, which should not be too high, since an osmotic medium with a low molecular weight can easily penetrate the cells of fruits and vegetables [42]. Furthermore, the concentration of the osmotic medium impacts mass transfer phenomena during osmotic dehydration [43]. It is important that the solvent chosen is effective in dehydrating the food, which leads to a fast rate of water loss from the food (WL), a low water activity value (aw), and the production of products with improved organoleptic characteristics without affecting their nutritional value, always at a low cost [44].

Recent trends in human nutrition have spurred the search for healthier solutions. Sucrose, glucose, and fructose syrups commonly used in osmotic dehydration can have adverse effects as they increase blood sucrose levels [43]. Although sodium chloride is a great osmotic agent for vegetables, its usage in concentrated fruit pieces is limited due to it imparting a salty taste to the food [42]. Therefore, efforts are being made to find alternative solutions that do not affect human health. The use of fruit juices or extracts from their by-products is becoming popular due to the abundance of bio-constituents and their potential to increase food quality [42].

Fruits and vegetables are sources of bioactive compounds, such as anthocyanins, carotenoids known for their physicochemical characteristics, nutritional value, and functional properties [45]. Anthocyanins, in particular, are a significant group of water-soluble natural pigments, with numerous health benefits [46]. The infusion of bioactive compounds through osmotic treatment into solid food matrices, without altering their physical structure, has been demonstrated in model food systems, including watermelon rind infused with anthocyanin [47]. The efficacy of bioactive compound infusion depends on the intercellular gaps within the natural food matrix [48]. Incorporating these compounds into food tissue can mitigate degradation reactions and enhance nutritional value [49].

Osmotic agents are presented in Table 2, alongside the food products that have been dehydrated.

**Table 2:** Osmotic agents used in the osmotic dehydration process.

Osmotic Agent	Food Product	Impact	Reference
Glycerol and inulin	Plums	The osmotic dehydration of plums resulted in water loss (WL) and water retention (WR) values of 30% and 29%, respectively, along with inulin (INU) and glycerol (GLY) concentrations of 119 mg/g and 373 mg/g, respectively. This indicates that osmotic dehydration effectively reduces the water content in the plums while enriching the product with beneficial compounds like inulin, thus enhancing the nutritional value and preparing the fruit for subsequent conventional drying processes.	[50]
Salt	Carrot Potato	It inhibits both oxidative and non-enzymatic browning. Further, facilitating mass transfer and preventing surface shrinkage makes it applicable as an osmotic agent—not, however, in fruit dehydration due to its salty taste.	[51,52]
Lactose	Zucchini	Lactose at 49.99% yielded optimum water loss, solid gain and dimensionless moisture content.	[53]
Sucrose	Quince	The osmotic dehydration of quince slices achieved significant mass reduction, water loss, and soluble solids gain, along with improved rehydration and colour metrics, demonstrating the effectiveness of the process.	[54]
Sodium chloride solution	Peas	Drying of fresh green peas subjected to osmotic pre-treatment at optimized conditions, followed by three-stage convective drying, would yield dried peas having low moisture content, minimum colour change, and sufficient sphericity and hardness.	[55]
Sodium chloride	Vegetables	Effectively slows down both oxidative and non-enzymatic browning. The bleaching effect on coloured products can be avoided by using combinations like salt and sugar. Shrinkage is also prevented.	[56]
Lactose	Apple	Exhibits a sweetness level significantly lower than sucrose and demonstrates limited solubility in aqueous solutions.	[56]

While current osmotic agents are effective, there is significant room for improvement in terms of health impacts and economic efficiency. The development of new osmotic agents, particularly those derived from natural sources like fruit juices, can address health concerns associated with traditional agents. Additionally, there is a need for continuing research into the molecular characteristics of osmotic agents and their interaction with different food matrices. By doing so, it may be possible to optimize the osmotic dehydration process further, enhancing the nutritional quality and safety of dehydrated food products while maintaining low production costs.

### 3.2. Temperature

Temperature is the most significant factor influencing the rate of mass transfer during osmotic dehydration. Elevated temperatures accelerate water loss, while solid gain is less affected [57]. This occurs because increased temperatures enhance the permeability of cell membranes, decrease the viscosity of the osmotic agent, and consequently facilitate water penetration into the product. An increase in temperature leads to enhanced water loss, thereby achieving water equilibrium through the diffusion of water molecules rather than solids. As the temperature rises, the diffusion of solid molecules through the cell membrane of the food becomes more challenging, resulting in a greater transport of water molecules from the fruit to the osmotic solution. Upon reaching equilibrium, the food will have absorbed only a minimal number of solids [58].

Moreover, given that the fruit sample is porous, a high temperature will also cause the release of trapped air from the tissue, enhancing the efficacy of osmotic pressure in removing water. This process not only improves the uptake of solids, but also the removal of water [59]. On the other hand, as the temperature increases, the viscosity of the osmotic solution decreases along with the external barrier to mass transfer at the product surface. As a result, this promotes higher rates of solute diffusion into the fruit and the efflux of water.

This ratio can be clearly seen in Equation (19) expressing the diffusion constant (*D_eff_*):

(19) $$Deff=Do\cdot \exp\left(-\frac{E_{a}}{RT}\right),$$

where *E_a_* is the activation energy (kcal/mol), *T* is the temperature (K), *Do* is the reference state diffusion constant (cm^2^/s), and R is the global gas constant = 8.314 (J/mol·K).

In osmotic treatment of autumn olive berries at temperatures ranging from 20 to 70 °C, the water loss and a_w_ were maximized at 70 °C, compared to treatments at ambient temperature [60]. The optimization of the osmotic dehydration process for mushrooms revealed that increasing the temperature to 50 °C yielded the best results. This optimal temperature facilitated efficient dehydration, improving the overall quality and characteristics of the mushrooms [61]. Studies have indicated that temperature impacts water loss kinetics, without affecting solid gain, particularly within the 30 to 60 °C range for fruits and vegetables [17]. At temperatures above this range, quality degradation phenomena, such as enzymatic browning and alterations in colour, aroma, and cell wall structure of the food, are often observed [62]. The optimum temperature for osmotic treatment varies according to the type of food being processed.

While temperature is a critical factor in osmotic dehydration, it must be carefully controlled to prevent quality degradation. Understanding the specific temperature thresholds for different types of food can optimize the process, ensuring maximum water loss without compromising the food’s sensory and nutritional attributes. Furthermore, there is a necessity for more detailed studies on the interaction between temperature and other osmotic dehydration parameters to develop more efficient and standardized processes.

### 3.3. Concentration of Osmotic Solution

The concentration of solute in the osmotic solution is a significant factor in osmotic dehydration. As the concentration of the osmotic agent increases, the water activity of the osmotic solution decreases, thereby enhancing water loss. Generally, a large osmotic potential difference between the solution and the sample leads to a high rate of solute and water diffusion [23]. Specifically, research on autumn olive berries has demonstrated that higher concentrations of osmotic solution (70%) resulted in increased osmotic pressure, which consequently led to greater uptake of solids and more extensive water loss throughout the osmotic process [60]. Increasing the concentration of osmotic agents during the osmotic dehydration of zucchini significantly enhanced both water loss and solid gain [50]. Similarly, the optimization of osmotic dehydration in quince revealed that increasing the sucrose concentration resulted in water loss reaching up to 25.77° Brix [52].

Furthermore, the swelling action of the membrane may facilitate greater mass transfer of sugar molecules with increasing concentration, potentially enhancing the permeability of the cell membrane. These findings suggest that selecting a medium with a higher concentration may offer advantages in terms of accelerating water loss and achieving a significantly higher gain of solids. However, other studies have indicated that using a high dosage of an osmotic agent might not necessarily improve solid gain. The decrease in mass transfer at maximum sucrose concentrations could be attributed to a case hardening effect, where the increased viscosity more significantly restricts the penetration of foreign liquids when the external solution is highly concentrated. Indeed, due to rapid concentration, external cell layers may become more rigid resulting in the case hardening effect [53].

Carefully balancing the concentration of the osmotic agent is crucial to optimizing both water loss and solid gain. While higher concentrations can enhance dehydration efficiency, they may also lead to issues such as case hardening, which can negatively impact the quality of the final product. Therefore, a comprehensive understanding of the concentration effects on different food matrices is essential for optimizing the osmotic dehydration process. Further research is needed to identify optimal concentration ranges that maximize dehydration efficiency without compromising the sensory and nutritional qualities of the food.

### 3.4. Ratio of Food to Osmotic Medium

An important factor in the osmotic dehydration of fruits and vegetables and in mass transfer phenomena is the ratio of food to the osmotic agent. This ratio is capable of creating the driving force for the diffusion of water molecules. As the osmotic dehydration progresses, the solution gradually becomes more dilute, leading to a reduction in the rate at which water molecules are removed from the food material.

Increasing the ratio offers certain advantages, notably in terms of minimizing the dilution of the osmotic solution. This, in turn, prevents a substantial decline in osmotic pressure during the process, thereby sustaining its effectiveness [54]. However, this benefit come with its own set of challenges. A higher ratio necessitates a larger volume of osmotic solution and intensifies the problem regarding its disposal and potential reuse. Consequently, many processing methodologies focus on smaller solution ratios, typically ranging between 1:2, 1:3, and 1:4. Not only do these ratios prove economically more viable, but they also mitigate the logistical complexities associated with managing larger volumes of the osmotic solution.

By striking a delicate balance between the ratio of food to osmotic agent, practitioners can optimize the osmotic dehydration process, ensuring both efficiency and sustainability in fruit and vegetable preservation techniques.

Carefully selecting the appropriate ratio of food to osmotic agent is essential for optimizing the dehydration process. A balanced ratio ensures that the osmotic pressure remains effective throughout the process, preventing significant dilution that could slow down water removal [20]. Additionally, using smaller solution ratios is not only more economical but also addresses practical issues such as disposal and reuse of the osmotic solution. There is a need for further research to determine the optimal ratios for different types of food, as well as to develop more sustainable practices for managing the osmotic solutions used in the dehydration process.

### 3.5. Duration of Osmotic Dehydration Process

The processing time, denoting the time that food remains immersed in the osmotic solution, stands as a crucial determinant in osmotic dehydration. Its extension correlates directly with the amplified moisture loss from the food material. With prolonged processing time, there is a corresponding increase in the quantity of water extracted from the food into the osmotic solution. Particularly noteworthy is the significance of the initial phase of processing, where rapid mass transfer phenomena occur, significantly influencing the subsequent course of evolution of the process.

Specifically, studies have revealed a pattern wherein mass transfer transpires at an accelerated rate within the initial 2 h, followed by a deceleration in drying rate [55]. The identification of the optimal treatment time becomes pivotal, representing the juncture where maximum water removal coincides with negligible uptake of solids [56].

Leahu et al. (2020) explored the osmotic dehydration of apple and pear slices in fructose solutions, finding that significant changes occurred over time [63]. Within the first hour, the rate of water loss decelerated substantially. By the end of three hours, the apple slices had shed a substantial amount of their initial moisture content and had gained a significant quantity of solids, indicative of sugar uptake. This study highlights the importance of understanding the kinetics of osmotic dehydration and the impact of time on water loss and solid gain. Understanding the dynamics of processing time not only facilitates efficient moisture removal but also offers insights into optimizing the quality attributes of osmotically dehydrated fruits and vegetables.

Determining the optimal processing time is essential for achieving efficient moisture removal while maintaining product quality. Prolonged processing can lead to excessive solid uptake, which might negatively affect the texture and taste of the product. Therefore, understanding the kinetics of mass transfer during the initial and subsequent stages of dehydration can help optimize the process, ensuring maximum water loss with minimal solid gain. This balance is crucial for producing high-quality osmotically dehydrated fruits and vegetables, which retain their desired sensory and nutritional attributes.

### 3.6. Agitation

Agitation plays a pivotal role in the osmotic dehydration process, significantly influencing the rate of mass transfer increases. Its efficacy lies in diminishing resistance by fostering continuous contact between the food material and the osmotic solution. This effect becomes particularly pronounced in case of solutions of higher concentration and viscosity. In addition, agitation serves to prevent the formation of a surface layer of solids on the exterior of the food product, thereby facilitating the unimpeded movement of water.

Reports indicate that the manner of agitation directly impacts the rate of water loss, with turbulent flow expediting the process compared to laminar flow [23]. However, it is important to note that while agitation enhances mass transfer, a potential drawback exists in its application: the risk of inadvertently damaging fragile food products.

Researchers have extensively examined the impact of agitation on osmotic dehydration. A recent study by Adekeye et al. (2020) extensively examined the impact of agitation on osmotic dehydration [64]. The research focused on the osmotic dehydration of garden egg (*Solanum aethiopicum*) using sodium chloride solutions at varying concentrations, temperatures, and agitation times. The study found that agitation significantly increased both water loss and solid gain compared to static conditions. Similarly, Ramya & Jain (2017) reviewed various factors affecting osmotic dehydration and highlighted that agitation during the process improves mass transfer rates, enhancing dehydration efficiency and product quality [13]. These findings underscore the importance of agitation in optimizing osmotic dehydration processes, emphasizing its role in improving the dehydration efficiency and overall quality of the dehydrated product.

Their findings highlighted a distinct advantage for agitated samples, showcasing a more significant reduction in weight and, consequently, water loss, when compared to their non-agitated counterparts.

Understanding the nuanced effects of agitation not only informs the optimization of osmotic dehydration processes but also underscores the delicate balance between enhancing mass transfer efficiency and safeguarding the integrity of food products.

### 3.7. Food Contact Surface with the Osmotic Medium

The interface between the food and the osmotic medium plays a critical role in determining the extent of water loss and solid uptake during osmotic dehydration [65]. Particularly noteworthy is the concept that, up to a certain ratio A/L (total area/half thickness), samples with a higher specific surface area tend to exhibit greater water loss and solid prevention compared to other geometries.

A recent study by Zecchi & Gerla (2020) underscores the significant influence of product size and shape on osmotic dehydration outcomes [66]. The research demonstrated that different shapes and sizes of tomato halves exhibited variations in water loss and solid gain during osmotic dehydration, with smaller and thinner samples showing higher water loss rates due to greater surface area exposure. Additionally, the study noted that cube-shaped samples tend to favour solid uptake over water loss, as the surface area-to-volume ratio plays a crucial role in the mass transfer process during dehydration. This highlights the pivotal role that surface characteristics play in determining the efficiency of osmotic dehydration, especially during shorter dehydration times, aligning with the understanding that dehydration primarily occurs due to convection phenomena operating at the surface. These findings emphasize the importance of considering the shape of food products when designing and optimizing osmotic dehydration processes to achieve the desired outcome [13].

By discerning the intricate relationship between food geometry and osmotic dehydration dynamics, practitioners can tailor processing parameters to optimize both water removal and solid absorption, thereby enhancing the overall quality of the final product.

### 3.8. Species, Variety and Maturity Level

The responses to osmotic dehydration exhibit significant variability across different plant species, varieties, and even within the same variety at different maturity levels [67]. This diversity in response stems from the intricate interplay of various factors that shape the natural tissue. Among these factors are species, variety, and maturity level, all of which influence critical aspects such as cell membrane structure, the ratio of protopectin to soluble pectin, the presence of insoluble solids, intercellular gaps, tissue compactness, and the presence of trapped air. The product and osmotic medium’s diffusional mass exchange is significantly impacted by these structural variations.

A recent study by [68] highlights the impact of structural variations in potatoes on the effectiveness of osmotic dehydration. The study found that different types of potatoes exhibited significantly different rates of water loss when subjected to identical osmotic dehydration conditions, with variations in water loss rates observed based on the structural differences of the potato tissues. This underscores the importance of understanding and accounting for the inherent variability in plant tissues when designing and optimizing osmotic dehydration processes [68,69].

## 4. Modern Pre-Treatment Applications or Methods

Pre-treatment methods, particularly nonthermal processing methods, have been proposed to enhance mass transfer [70]. These methods play a crucial role in preparing food materials for osmotic dehydration, facilitating efficient moisture removal and solid uptake. Some popular pre-treatment methods are analysed below.

### 4.1. Pulsed Electric Fields (PEF)

Pulsed electric fields (PEF) as a pre-treatment method before osmotic dehydration present several benefits in the context of fruit processing. Applying PEF causes cell membranes to permeabilize reversibly, improving mass transfer during the subsequent osmotic dehydration processes [71]. This enhanced permeability allows for more efficient extraction of intracellular compounds, thereby increasing product quality. Research has shown how PEF improve the uptake of water and other solutes during osmotic dehydration, reduce tissue resistance, and modify cell structure. PEF pre-treatment is a desirable method for maximizing fruit processing efficiency because it has also been linked to a decrease in processing time [72].

The PEF method involves exposing fruit tissues to brief, powerful electric pulses, typically in the order of microseconds to milliseconds. The cellular structure is not irreversibly damaged by the regulated application of electric fields, which cause permeabilization of cell membranes. The effectiveness of PEF as a pre-treatment technique is contingent upon a number of critical factors, such as the number of pulses, field strength, and pulse duration. To attain the appropriate degree of permeabilization without sacrificing the fruit’s overall quality, it is imperative to optimize these characteristics [73]. The optimum pre-treatment conditions are revealed by the effects of PEF on distinct fruit quality parameters at different processing stages [74].

Studies indicate that that PEF treatment before osmotic dehydration had a positive impact on mass transfer of fruits. Regarding strawberries dehydrated in sucrose and trehalose solution, the application of the lowest electric field intensity (100 V cm^−1^) was already sufficient to increase water loss by 12% and 6%, respectively, after one hour of osmotic dehydration, partially preserving the cell viability and maintaining the fresh-like characteristics of fruits [75]. PEF application prior to osmotic dehydration of apples enhanced water loss during the process. In particular, the use of PEF at a field strength of 5 kV/cm and 10 pulses resulted in the highest efficiency ratio for osmotic dehydration [76,77].

Other studies regarding blueberries treated with PEF at 2 kV/cm indicated a notable reduction in osmotic dehydration time from 120 to 48 h, achieving the target moisture content of 3.0 g/g initial dry matter. Additionally, PEF-pre-treated blueberries exhibited lower microbial populations, indicating enhanced microbiological quality [77]. Bell peppers pre-treated with PEF presented enhanced cell membrane permeabilization with increasing field strength and pulse number, leading to higher initial drying rates compared to untreated samples. Also, effective water diffusivity (D_eff_) values were notably higher for PEF pre-treated samples, indicating improved water diffusion characteristics [78].

To achieve the desired effects on cell permeability, PEF pre-treatment conditions prior to osmotic dehydration require careful consideration of elements like electric field strength, pulse duration, and treatment time. Understanding the relationship between process parameters and outcomes is crucial, according to research. Combining ideal PEF pre-treatment conditions with osmotic dehydration afterwards is a calculated tactic to raise the productivity and quality of fruit processing [79].

### 4.2. Ohmic Heating (OH)

Ohmic heating is a promising pre-treatment method before osmotic dehydration in fruit processing and can offer various notable benefits in terms of enhancing mass transfer kinetics and improving overall product quality. It is an electrothermal process that involves the passage of electricity through a food material, generating heat internally, and leading to rapid and uniform heating [80]. Thus, the product behaves as an electrical resistor. Ohmic heating can be used as a pre-treatment method in order to increase tissue permeability (by solubilizing the pectic substances that make up the cellular wall and by causing cell membranes to electroporate) and facilitate improved water and solid transfer during subsequent osmotic dehydration [81]. The osmotic dehydration process can be enhanced using ohmic heating as a pre-treatment, resulting in reduced processing times and improved product quality [81].

The ohmic heating method is based on the application of electricity directly through the fruit material, which produces heat due to its electrical resistance. The electrical conductivity of the product and the current caused by the voltage gradient in the field are directly correlated with the heat produced inside the food [53]. By ensuring a uniform temperature distribution and minimizing thermal gradients, in-situ heating lowers the possibility of localized over-processing or underprocessing. Voltage, current density, treatment time, and other important factors determine how effective ohmic heating is as a pre-treatment approach. Optimization of these conditions is essential to achieve the desired level of tissue permeability without compromising the overall quality of the fruit.

To accomplish the intended effects on fruit tissue, the conditions for ohmic heating as a pre-treatment before osmotic dehydration require careful consideration of electrical factors. Research by [82] explores the optimization of ohmic heating in fruit processing, emphasizing its impact on bioactive compounds and antioxidant activity. This scientific exploration sheds light on the importance of understanding the interplay between process parameters and outcomes, guiding the development of efficient and sustainable fruit processing methodologies. The integration of ohmic heating as a pre-treatment method with subsequent osmotic dehydration represents a promising avenue for improving the quality and efficiency of fruit processing operations.

The utilization of ohmic heating as a novel approach for osmotic dehydration of quince demonstrated significant improvements in drying kinetics and quality attributes. The study identified optimal parameters—40 V/cm electrical field intensity, 30 min holding time, 16.67% sucrose concentration, and 270 W microwave power—that notably enhanced dielectric characteristics, total phenolic content, rehydration ratio, and colour preservation compared to control samples [83]. Additionally, ohmic heating-induced blanching of apple tissues induced significant alterations in cellular structure, thereby improving mass transfer during subsequent osmotic dehydration [84]. Similarly, the application of ohmic heating to strawberries resulted in a marked increase in mass transfer and effective diffusion rates, facilitating enhanced transfers of sugar and water during the osmotic dehydration process [85]. These findings underscore the efficacy of ohmic heating as a pre-treatment method to improve mass transfer kinetics and quality attributes during osmotic dehydration.

### 4.3. Ultrasound (UP)

In fruit processing, the use of ultrasound as a pre-treatment technique prior to osmotic dehydration is a novel strategy with numerous advantages for food safety and industrial uses. High-power ultrasonic waves at low frequencies (20–100 kHz) are supplied sparingly at low temperatures to cause quick expansions and compressions that remove moisture and create a sponge-like effect, having a dual impact on both water loss and solid gain. According to [12,86], this mechanism produces tiny channels that allow osmotic fluids to enter the intercellular spaces of partially dried materials. These passageways provide water with an easier path to disperse toward the surface of the fruit. Notably, Nowacka et al. (2014) discuss how the adaptability of ultrasound allows for the development of both low- and high-intensity waves, which are useful for non-invasive analytical techniques and accelerating heat and mass transfer processes in the food sector, respectively [36].

In order to minimize food deterioration, the combination of ultrasound and osmotic dehydration is conducted at ambient temperature. Carried out even at low temperatures, it achieves high rates of solid gain and water loss which improves the preservation of natural colour, scent and nutrient content. However, depending on the type of ultrasound sequence used (continuous or pulsed) and the characteristics of the pulses (length, intensity and frequency), the cavitation effect created removes strongly attached moisture, but can also lead food tissue to experience local heating [36].

A 3-h ultrasonic treatment during osmotic dehydration of kiwifruit enhanced water loss and significantly increased the uptake of solids within the fruit tissue compared to samples not treated with ultrasound, leading to improved dehydration efficiency and product quality. The subject of this investigation is high-intensity ultrasound, which has a variety of uses in the food business and causes permanent changes in material structure. High-intensity ultrasound damages materials and initiates a variety of chemical reactions, including oxidation processes, according to Nowacka’s thorough study from 2021 [9]. It works well for cleaning machines, separating intracellular proteins, and speeding up heat and mass transfer processes. Immersion ultrasonography is frequently used to expedite osmotic dehydration prior to and during fruit processing [36,87]. There is potential for improving the effectiveness and quality of fruit processing processes through the use of ultrasound as a pre-treatment technique.

The combined results of multiple researches examining pre-treatments with ultrasound prior to osmotic dehydration of diverse fruits show notable improvements in drying behaviour and quality attributes. In comparison to untreated groups, strawberries that underwent ultrasound-assisted osmotic dehydration exhibited shorter osmotic dehydration periods, as well as enhanced water loss and solid gain [88]. In persimmons, the ultrasound pre-treatment significantly reduced the overall drying time of melons and raised the effective water diffusivity. Furthermore, compared to untreated groups, ultrasound-assisted osmotic dehydration significantly improved the rate of rehydration and the total phenolic content in persimmons without significantly altering their colour [89].

Moreover, ultrasound pre-treatment led to the formation of microchannels and an increase in the average cross-section area of cells, which facilitated water redistribution across cellular substructures during the osmotic dehydration of kiwifruits [90]. This positively influenced mass exchange during the process. All of these results highlight how well ultrasound-assisted osmotic dehydration works to improve drying kinetics, shorten processing times, and improve the general quality of dried fruits [89].

In conclusion, ultrasound is a useful instrument that can be used in high-intensity processes for a variety of results in the food sector. It can also cause microscopic changes in fruit tissues, improving mass transfer. The effectiveness of the immersion method in expediting osmotic dehydration before and during the process highlights the usefulness of ultrasound in optimizing fruit processing operations. These advancements represent a major step forward in the search for effective, superior fruit processing techniques [9].

In Table 3, the key features, benefits, mechanisms, key factors, and challenges of Pulsed Electric Fields (PEF), Ohmic Heating (OH), and Ultrasound (UP) are summarized to provide a clear comparison of these pre-treatment methods.

## 5. Application of Osmotic Dehydration in Food Processing

Osmotic dehydration plays a crucial role in various aspects of food processing, particularly in the preservation and enhancement of food products. Osmotic dehydrated products, characterized by their stability and intermediate-moisture content, serve as ready-to-eat options. This method finds wide application across industries, including dairy, confectionery, fruit, vegetable, and bakery sectors as well as in the production of concentrates and jams.

Implementation of such processes on strawberries resulted in increase of certain compounds and loss of natural fragrance components that led to high sensory acceptability [91]. Significantly, breakfast cereals used osmotically dried quince that was submerged in different concentrations of sucrose solutions at various temperatures where the final product’s colour, water activity, vitamin C content and texture were greatly impacted [92]. The creation of ready-to-eat snacks requires dipping of the fruits in solution of sucrose to reduce their weight, resulting in the development of intermediate-moisture food products, or candied fruits with enticing colour and texture [93].

The impact of osmotic dehydration extends beyond mere preservation, as it significantly enhances certain nutritional, functional, and organoleptic properties of the product. Operating at room temperature, the process minimizes damage to flavour and colour, while the high sugar concentration surrounding fruits and vegetables prevents discolouration. Researchers have explored different osmotic agents and concentrations to optimize this technique, aiming to maintain the nutritional quality and sensory attributes of the processed foods [22].

Furthermore, osmotic dehydration enables the reimagining and improvement of conventional processes. Innovative techniques have been developed, such as the production of fragrant concentrates from fruits and vegetables. These fruits undergo an osmotic preconcentration stage before being crushed, processed, pasteurized, or frozen to create these concentrates. Similarly, a single procedure has the potential to streamline the conventional salting/drying sequence in fish processing [20].

Moreover, osmotic dehydration is harnessed to produce premium food items like osmotically dehydrated jams and jellies. By selectively removing water from fruits and allowing sugar and flavourings to diffuse into the cells, this method contributes to achieving the desired texture and sweetness. A concentrated syrup forms around the fruit particles through the regulated osmotic process, enhancing sensory qualities and extending shelf life. This method has been extensively researched and has been employed to produce a wide array of fruit-based products, offering valuable insights into optimizing osmotic dehydration conditions for diverse fruits and producing high-quality products [22].

Expanding the exploration of osmotic dehydration’s applications and refining its techniques continue to pave the way for innovation in food processing, ensuring the production of superior-quality products that meet consumer demands for taste, nutrition, and convenience.

## 6. Comparison with Other Drying Methods

Osmotic dehydration is widely recognized for its ability to maintain food quality compared to other, more intensive dehydration methods such as freeze-drying and hot air drying. This advantage is particularly evident for fruits and vegetables, which are susceptible to tissue damage when exposed to high temperatures. Osmotic dehydration offers several benefits in this regard. It inhibits enzymatic browning and preserves natural colour without the need for additives like sulfur compounds. Additionally, it often enhances the taste and achieves a desirable texture in the final product offering a significant quality advantage over other dehydration techniques [12].

One of the primary benefits of osmotic dehydration is its reduced energy consumption. Unlike freeze-drying, which requires significant energy input for phase changes from liquid to solid, osmotic dehydration removes moisture through physical diffusion without requiring the latent heat of evaporation. This results in lower energy requirements and simpler equipment, making osmotic dehydration a cost-effective option [23].

Furthermore, studies by [20] have demonstrated that partial dehydration and solute uptake during osmotic dehydration can prevent structural collapse during subsequent drying processes. This finding underscores the importance of osmotic dehydration as a preparatory step in food processing, contributing to the overall quality and stability of the final product [94].

Logistically, osmotically dehydrated products offer advantages due to their reduced weight and volume facilitating easy handling and cost-effective transportation. Also, the increase of products’ shelf life in storage, achieved using simple and low-cost equipment, offers economic benefits. This is particularly beneficial for industries looking to minimize storage and distribution costs while maintaining high product quality [20,95].

Despite its advantages, osmotic dehydration has certain limitations that necessitate careful consideration. The process can lead to potential weakening of the product’s flavour due to acidity reduction and may result in textural and colour alterations. The sugar coating formed during the process may not always be in line with aesthetic tastes and may need to be removed promptly to meet consumer preferences [20].

Furthermore, operational complexities may arise when scaling up the osmotic process, necessitating meticulous control over factors such as uniformity and consistent product quality. High concentrations of osmotic solutions are often required, leading to increased operational costs and posing challenges in managing the disposal of concentrated solutions, thus contributing to environmental concerns [13,20,23].

The energy consumption in osmotic dehydration is primarily influenced by the prolonged processing times needed to achieve the desired moisture content, which can inadvertently increase overall energy usage, if not properly managed. To optimize energy efficiency, several key parameters such as temperature, osmotic agent concentration, and agitation must be carefully controlled [20]. For instance, elevating the process temperature can accelerate the dehydration rate but might also increase the risk of thermal degradation of the product’s quality attributes [94].

In conclusion, osmotic dehydration stands out as a versatile and cost-effective method for preserving food quality, particularly in fruits and vegetables. Its ability to inhibit enzymatic browning, preserve natural colour, enhance taste, reduce energy consumption, and improve structural integrity makes it a valuable technique in food processing. Additionally, the logistical advantages associated with osmotically dehydrated products further contribute to its appeal in the food industry. While there are challenges associated with osmotic dehydration, such as potential flavour alterations and operational complexities, ongoing research and technological advancements are continually addressing these issues, ensuring the method remains a viable and effective option in the food industry [4,20].

In Table 4, the key aspects of different dehydration methods are presented, highlighting their advantages and disadvantages.

## 7. Future Developments in Osmotic Dehydration

Future advancements in osmotic dehydration are expected to focus on enhancing the technological components of the process. Research initiatives will likely explore innovative techniques to increase mass transfer kinetics, involving the introduction of novel osmotic agents, improved equipment designs, or the integration of cutting-edge technology such as electric fields or ultrasound. A deeper understanding of the molecular interactions between the osmotic solution and food matrix could lead to the development of more efficient and long-lasting osmotic dehydration methods. Combining computer modelling and simulation methods with experimental data may yield important insights into process parameter optimization and the effects of many factors on the overall effectiveness of osmotic dehydration [22,96,97].

Research efforts should prioritize maintaining the sensory qualities and nutritional value of osmotically treated products. Investigating the impact of osmotic dehydration on the retention of vitamins, antioxidants, and bioactive substances in food materials is crucial. Optimizing osmotic conditions to prevent the degradation of heat-sensitive nutrients and preserve the colour, texture, and flavour of end products is paramount. This may involve studying the effects of the osmotic process on cell structure and developing mitigation techniques to counter any adverse effects on the overall food quality [96,98].

Sustainability concerns and waste reduction during the osmotic dehydration process also warrant attention in future studies. While osmotic dehydration has primarily been conducted on a pilot scale, economic and environmental issues arise from the use of extremely concentrated solutions. Research efforts may focus on exploring sustainable methods and eco-friendly osmotic agents to mitigate negative environmental impacts. Adopting a more circular and sustainable strategy could involve investigating the value-adding potential of by-products generated from osmotic dehydration, such as extracted chemicals or discarded osmotic solutions. Additionally, exploring alternative recycling mechanisms, such as filtration, to manage high concentration levels is essential. Further research may concentrate on maximizing the energy efficiency of osmotic dehydration procedures to reduce overall resource usage and enhance the technique’s commercial viability on an industrial scale [99,100].

Innovative approaches to improve energy efficiency in osmotic dehydration are emerging. Incorporating renewable energy sources, such as solar or geothermal energy, into the dehydration process presents a promising avenue for reducing the environmental footprint. Advanced process integration techniques, such as using heat exchangers to recover and reuse thermal energy, can further minimize energy consumption [20].

Several practical strategies can be employed to optimize osmotic dehydration, enhancing both efficiency and product quality. The use of eco-friendly osmotic agents, such as plant-based extracts or natural sugars, is gaining attention for their lower environmental impact and potential health benefits [20]. Additionally, pre-treatment methods such as those mentioned above can significantly enhance mass transfer rates, reducing processing times and improving the dehydration efficiency [101]. These methods disrupt cell membranes, facilitating more efficient water removal and solute uptake without compromising nutritional and sensory qualities [23].

Optimizing the solution-to-sample ratios is another critical factor for improving osmotic dehydration efficiency. Studies have demonstrated that maintaining an appropriate ratio ensures sufficient osmotic driving force throughout the process, which is essential for achieving uniform dehydration and high quality. Additionally, cyclic operation, where the osmotic solution is periodically replaced or rejuvenated, can maintain the driving force for dehydration, thus shortening the overall processing time [23].

In conclusion, future research in osmotic dehydration has the potential to drive innovation, improve sustainability, and enhance the overall quality and efficiency of food processing operations. By addressing these challenges and opportunities, future research in osmotic dehydration has the potential to drive innovation, improve sustainability, and enhance the overall quality and efficiency of food processing operations.

## 8. Conclusions

Osmotic dehydration stands out as an effective drying method for food, capable of reducing weight by up to 50%, while simultaneously enhancing shelf life and food quality. This energy saving technique minimally impacts the nutritional and sensory properties of the food product, making it a preferred choice in food processing. Additionally, osmotic dehydration requires minimal equipment and can be an economical drying process, further adding to its appeal.

Despite being a commonly utilized strategy for several decades, osmotic dehydration continues to hold high potential for further development and refinement. Numerous advancements can be made to increase its effectiveness and applicability in various food processing scenarios. The widespread applications in the food business highlight its popularity and significance in the industry.

Moving forward, continued research and innovation in osmotic dehydration are expected to unlock new possibilities and optimize existing processes. These advancements will not only enhance the efficiency and effectiveness of osmotic dehydration but also contribute to the continued improvement of food preservation and processing techniques.

## Figures and Tables

**Table 1:** Comparison of osmotic dehydration models based on key parameters and outcomes.

Model	Applications	Advantages	Limitations	References
Azuara	Effective for a wide range of food products such as apples, bananas, kiwifruit, cherry tomatoes, and goat meat.	Flexibility in fitting experimental data without geometric or specific process condition restrictions.	Relies on experimental data within a specific range for accurate evaluation.	[24]
Page	Suitable for thin layers of material such as apples, bananas, cherry tomatoes, and kiwifruit.	Straightforward and fits well with thin material layers.	May not be suitable for thicker or more complex structures.	[25]
Panagiotou	Effective for sea bass fillets, beef meat, and carrots.	Takes into account multiple influencing factors, providing versatile applicability.	Requires detailed knowledge of various influencing factors, which can be complex to determine.	[26]
Crank	Used to approximate diffusion coefficients for apple, banana, beetroot, and potato.	Provides a theoretical foundation for diffusion.	Requires precise parameterization and assumptions that may not always hold in practical scenarios.	[27]

**Table 3:** Comparison of pre-treatment methods for osmotic dehydration.

Aspect	Pulsed Electric Fields	Ohmic Heating	Ultrasound
Benefits	Efficient mass transfer	Enhanced mass transfer	Improved mass transfer
	Improved product quality	Uniform heating	Faster drying
	Reduced processing time	Reduced processing time	Preservation of colour and nutrients
Mechanism	Membrane permeabilization	Internal heating due to electrical resistance	Creation of microchannels
	Enhanced intracellular compound extraction	Permeabilization of cell membranes	Enhanced water and solid transfer
Key Factors	Electric field strength	Voltage	Frequency
	Pulse duration	Current density	Intensity
	Number of pulses	Treatment time	Type of ultrasound (continuous or pulsed)
Challenges	Optimization of pulse parameters	Optimization of electrical conditions	Potential local heating
	Potential product damage	Potential product damage	Optimization of ultrasound parameters
	High energy consumption	Energy consumption	Potential structural damage

**Table 4:** Comparison of dehydration methods: key advantages and disadvantages.

Method	Advantages	Disadvantages
Osmotic Dehydration	Maintains high product quality (colour, taste, texture)	Potential flavour and texture changes
	Inhibits enzymatic browning without additives	Sugar coating may not meet aesthetic preferences
	Reduced energy consumption compared to freeze-drying	Scaling up may present operational complexities
	Lower energy requirements and simpler equipment	High osmotic solution concentrations can lead to increased operational costs and environmental concerns
	Improved structural integrity of products during subsequent drying	Prolonged processing times can increase overall energy usage if not managed efficiently
	Logistical benefits: reduced weight and volume, longer shelf life, cost-effective transportation	
Freeze-Drying	Excellent preservation of taste, texture, and nutrients	High energy consumption due to phase changes from liquid to solid
	Very low moisture content in final product	Expensive equipment and operational costs
Hot Air Drying	Simple and widely used technology	Can cause significant loss of nutrients and flavour
	Lower initial equipment cost	High energy consumption and potential for product damage due to high temperatures
	Faster processing time compared to freeze-drying	May require additives to prevent browning and preserve colour
